# Evaluation of serum alpha-1B glycoprotein and C-reactive protein levels as biomarkers of canine benign prostatic hyperplasia

**DOI:** 10.14202/vetworld.2025.1540-1548

**Published:** 2025-06-15

**Authors:** Grisnarong Wongbandue, Sekkarin Ploypetch, Piyada Pruksakitcharoen, Kittithee Udomrit, Kanisa Nujan, Rinrada Seekhumtae, Tanapron Thubthim, Nawarus Prapaiwan

**Affiliations:** 1 Department of Clinical Sciences and Public Health, Faculty of Veterinary Science, Mahidol University, Phuttamonthon, Nakhon Pathom, Thailand; 2 Faculty of Veterinary Science, Mahidol University, Phuttamonthon, Nakhon Pathom, Thailand

**Keywords:** alpha-1B glycoprotein, benign prostatic hyperplasia, canine biomarkers, castration, C-reactive protein

## Abstract

**Background and Aim:**

Benign prostatic hyperplasia (BPH) is a prevalent disorder in aging male dogs, characterized by prostate enlargement secondary to hormonal dysregulation and chronic inflammation. Identifying non-invasive biomarkers is crucial for improving diagnosis and monitoring therapeutic interventions. This study aimed to evaluate serum alpha-1B glycoprotein (A1BG) and C-reactive protein (CRP) concentrations in dogs with BPH before and after castration, to assess their diagnostic and prognostic utility.

**Materials and Methods:**

A total of 20 male dogs were assigned to two groups: healthy controls (n = 10) and BPH-affected dogs (n = 10). Blood samples were collected from controls and the BPH group at diagnosis and 1 month post-castration. Serum A1BG and CRP concentrations were measured using enzyme-linked immunosorbent assay and fluorescence immunoassay, respectively. Prostatic volume (PV) was evaluated ultrasonographically.

**Results:**

Dogs with BPH demonstrated significantly lower serum A1BG concentrations before castration compared to healthy controls (p < 0.01) and post-castration (p < 0.01). Post-castration A1BG levels were comparable to controls, suggesting biochemical normalization. Serum CRP concentrations remained within the normal range (<30 mg/L) across all groups and showed no significant differences. A significant negative correlation was observed between age and A1BG concentration in the pre-castration BPH group (r = −0.74, p = 0.02). Castration resulted in a marked reduction in PV, consistent with therapeutic response.

**Conclusion:**

Serum A1BG demonstrated potential as a sensitive biomarker for the diagnosis and therapeutic monitoring of canine BPH, in contrast to CRP, which exhibited limited diagnostic value. Normalization of A1BG levels post-castration supports its role in reflecting disease resolution. Integrating A1BG assessment into veterinary diagnostic workflows could enhance early detection, monitoring, and management strategies for BPH, offering a non-invasive and clinically informative approach. Further longitudinal studies with larger cohorts are warranted to validate these findings and explore long-term biomarker dynamics.

## INTRODUCTION

Benign prostatic hyperplasia (BPH) is a common age-associated disorder in intact male dogs, character-ized by epithelial and stromal proliferation leading to prostatic enlargement [1]. Hormonal imbalances, particularly an increased conversion of testosterone to dihydrotestosterone (DHT), a potent stimulator of prostatic growth, underpin its pathogenesis [2]. Chronic inflammation and oxidative stress further contribute to disease progression, with additional influences from body weight and breed predisposition [3, 4]. Clinically, BPH manifests with signs such as tenesmus, dysuria, pollakiuria, hematuria, urethral discharge, and complications including hematospermia, cystitis, constipation, and hindlimb pain [5]. Given its high prevalence, early diagnosis is essential for improving quality of life and longevity [6].

Diagnosis traditionally involves clinical history, rectal palpation, and imaging modalities such as radiography and ultrasonography, complemented by fineneedle aspiration cytology [1]. While histopatho-logical confirmation remains the gold standard, its invasiveness and associated risks, including acute prostatitis and peritonitis, limit its routine use [7, 8]. Non-invasive imaging techniques primarily evaluate prostatic size and structure [9], although additional diagnostic confirmation is often necessary [10, 11].

Management strategies for BPH encompass medical and surgical options. Pharmacological agents such as finasteride and osaterone acetate are effective, particularly in breeding animals, but are contraindicated in cases of testicular neoplasia [12, 13]. Castration remains the most definitive treatment for non-breeding dogs, significantly reducing prostatic volume (PV) through suppression of testosterone production [14]. However, anesthetic and surgical risks escalate in older dogs with concurrent comorbidities [15].

Recent research has prioritized the development of alternative diagnostic methods, particularly the identification of serum biomarkers [16]. Among these, canine prostate-specific arginine esterase has emerged as a promising candidate, reflecting androgenic stimulation and exhibiting significant alterations in BPH [17, 18]. C-reactive protein (CRP), an acute-phase protein, has also been investigated; however, Lehrer *et al*. [19] revealed no significant differentiation between BPH and prostate cancer based on CRP levels. In addition, alpha-1B glycoprotein (A1BG), implicated in inflammatory and platelet-related processes, has garnered attention due to its differential expression in BPH and its potential as a diagnostic and therapeutic biomarker [20–22].

Although imaging modalities and clinical evaluations remain the primary tools for diagnosing BPH in dogs, they have inherent limitations in terms of sensitivity, specificity, and invasiveness. Histopathological confirmation, although definitive, carries procedural risks and is not suitable for routine clinical use. Serum biomarkers, such as prostate-specific arginine esterase and CRP, have been explored; however, their diagnostic performance remains inconsistent, with CRP demonstrating limited utility in distinguishing BPH from other prostatic diseases. Emerging evidence suggests that A1BG may play a role in the pathophysiology of prostatic disorders, yet its clinical relevance as a biomarker for BPH diagnosis and therapeutic monitoring remains poorly characterized. Current studies on A1BG in canine BPH are sparse, predominantly descriptive, and lack evaluation of post-treatment dynamics, thereby limiting their translational application in veterinary practice.

This study aimed to evaluate the diagnostic and prognostic potential of serum A1BG and CRP concentrations in male dogs diagnosed with BPH. Specifically, we investigated the changes in serum A1BG and CRP levels before and after castration to determine their suitability as non-invasive biomarkers for disease detection and therapeutic monitoring. By elucidating the relationship between biomarker levels, PV changes, and castration status, this study aimed to provide foundational evidence for integrating serum A1BG measurements into clinical workflows to enhance the diagnosis, management, and follow-up of canine BPH.

## MATERIALS AND METHODS

### Ethical approval

All animal procedures were reviewed and appro-ved by the Institute Animal Care and Use Committee of the Faculty of Veterinary Science, Mahidol Univer-sity (Protocol No. MUVS-2023-09-58) in accordance with institutional and national guidelines for animal welfare. These procedures adhered to local legislation and institutional policies, and informed consent was obtained from all animal owners involved in the study.

### Study design and location

This study was conducted between December 2022 and December 2023 at Prasu-Arthorn Animal Hospital, Faculty of Veterinary Science, Mahidol University, Nakhon Pathom, Thailand.

### Animals and experimental grouping

A total of 20 client-owned male dogs scheduled for elective castration were enrolled. Eligibility was confirmed through comprehensive clinical examination, hematological analysis, and ultrasonographic assessment. Sample size determination was performed using G*Power version 3.1 software (Heinrich Heine University, Düsseldorf, Germany). An a priori power analysis was conducted, employing an independent t-test (power = 0.95, α = 0.05) and an assumed effect size (Cohen’s d) of 1.2 to calculate the required sample size.

**Figure 1:**
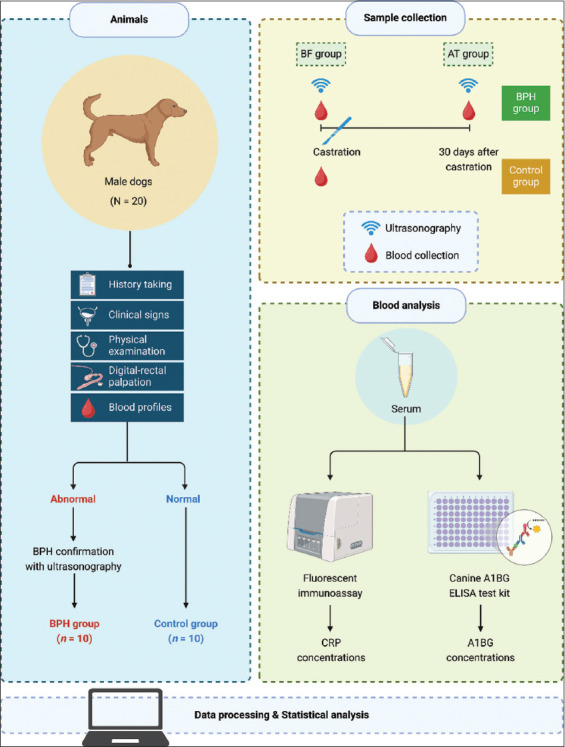
Schematic workflow of the experimental study design [This figure was created with BioRender, https://BioRender.com/f36u519].

The experimental design is depicted in Figure 1. Dogs were divided into two groups based on clinical and ultrasonographic findings: The healthy control group (n = 10) and the BPH-affected group (n = 10). The control group consisted of clinically healthy dogs aged 1–3 years, with body weights ranging from 3.9 to 30 kg. The BPH group consisted of dogs aged 2–8 years, with body weights ranging from 4 to 30 kg. The breed distributions for both groups are detailed in Table 1.

**Table 1:** Breed distribution of dogs in the control and BPH groups.

Breed	Number of dogs (n)	

	Control group	BPH group
Mongrel	7	1
Pomeranian	2	2
Chihuahua	1	1
Shih Tzu	0	2
American bully	0	1
Golden retriever	0	1
Jack Russell Terrier: A Jack Russell Terrier	0	1
Thai Bangkaew	0	1

BPH=Benign prostatic hyperplasia

### Diagnostic criteria and ultrasonographic evaluation

Dogs in the control group were selected based on clinical and laboratory findings. They exhibited no signs of prostatic disease, such as tenesmus, hematuria, or abnormalities upon rectal palpation, and had hematological and biochemical profiles within reference ranges. Dogs with any history of systemic illness, hormonal therapy, or abnormal screening results were excluded from the study.

Dogs diagnosed with BPH typically present with clinical signs, including sanguineous preputial or urethral discharge, hematuria, tenesmus, and difficulty in defecation [23]. A digital rectal examination revealed that a normally consistent, non-painful prostate, and blood tests showed no systemic abnormalities. Ultrasonographic evaluation was the primary diagnostic modality used to confirm BPH. Transabdominal ultrasonography was performed using a linear transducer (GE LOGIQ P6, GE HealthCare, USA). Diagnostic criteria included increased echogenicity or hyperechoic background, regular or coarse parenchymal stippling, homogeneous or heterogeneous parenchymal appearance, and the presence of small cysts or mineralized opacities [23, 24]. Dogs with hypoechoic or anechoic cavities larger than 0.5 cm, indicative of prostatic cysts or abscesses, were excluded from the study.

Prostatic dimensions were obtained by measuring the length, width, and dorsoventral diameter. PV was calculated using the formula:

PV (cm³) = (width × length × diameter) ÷ 2.6 + 1.8,

whereas estimated volume (EV) was determined as:

EV (cm^3^) = 0.33 × body weight (kg) + 3.28 [25].

Ultrasonographic assessments for the BPH group were conducted at 2-time points: At the time of BPH diagnosis and 1 month following castration (Table 2). Blood samples were collected from control dogs 1 day before castration and from BPH dogs at diagnosis (BF, referring to dogs before castration) and 1-month post-castration (AT, referring to dogs after castration).

**Table 2:** Summary of the estimated prostatic volumes, measured volumes before and after castration, and corresponding ratios.

ID	Age (years)	Estimated volume (cm^3^)	Prostatic volume before castration (cm^3^)	The ratio of the measured prostatic volume to the estimated volume	Prostatic volume after castration (cm^3^)
BP1	2	13.18	33.80	2.56	7.70
BP2	7	6.09	7.80	1.28	3.60
BP3	4	8.73	9.09	1.04	3.00
BP4	4	5.56	8.97	1.61	4.50
BP5	6	4.60	6.30	1.37	4.30
BP6	7	5.16	5.35	1.04	2.57
BP7	8	18.13	24.70	1.36	9.47
BP8	6	4.90	7.20	1.47	3.56
BP9	6	5.69	10.6	1.86	3.66
BP10	7	11.20	43.60	3.89	9.30

BP=Dog with benign prostatic hyperplasia

### Blood collection and sample processing

Peripheral blood samples (3 mL) were collected aseptically from the cephalic vein into plain tubes. Samples were allowed to clot at 25°C and subsequently centrifuged at 1,000 × *g* for 3 min. The serum was separated and stored at −20°C for up to 6 months before biomarker analysis.

### Biomarker analyses

#### CRP analysis

Serum CRP concentrations were quantified using a fluorescence immunoassay (Vcheck Canine CRP 2.0 Test Kit; Bionote, South Korea) following the manufacturer’s instructions. The assay’s intra- and inter-assay variability parameters were within acceptable limits for canine serum. Briefly, 5 μL of serum was diluted with 4 mL of diluent buffer, and 100 μL of the diluted sample was transferred into the test cartridge. The CRP concentration was measured after 5 min using the V200 Analyzer (Bionote, South Korea). A serum CRP concentration exceeding 30 mg/L was considered abnormal.

#### A1BG analysis

Serum A1BG concentrations were measured using a sandwich enzyme-linked immunosorbent assay (ELISA) kit specific for canine A1BG (MBS1602498; MyBioSource, San Diego, CA, USA), according to the manufacturer’s protocol. The assay detection range was 1–400 ng/mL, with intra-assay and inter-assay coefficients of variation reported as <8% and <10%, respectively. The ELISA employed a pre-coated plate containing a canine A1BG antibody. Standards and serum samples were added to the wells, followed by a biotinylated A1BG antibody. After incubation at 37°C for 60 min, streptavidin-horseradish peroxidase and tetramethylbenzidine substrates were sequentially added. Following a final 10 min incubation at 37°C in the dark, the reaction was terminated with sulfuric acid. Optical density was measured at 450 nm using a microplate reader.

### Statistical analysis

All statistical analyses were conducted using IBM Statistical Package for the Social Sciences Statistics version 18.0 (IBM Corp., Armonk, NY, USA). Data normality was assessed using the Shapiro–Wilk test and homogeneity of variance was evaluated with Levene’s test. For normally distributed data, between-group comparisons were performed using independent sample t-tests, whereas paired sample t-tests were used for pre- and post-castration comparisons. Correlations between variables were assessed using Spearman’s rank correlation coefficient. p < 0.05 was considered statistically significant.

## RESULTS

### Age and PV in the BPH group

The mean age of the dogs in the BPH group was higher than that of the control group, consistent with the age-related development of the condition. PVs were markedly larger in the BPH group before castration and demonstrated a significant reduction following the surgical intervention. Detailed data on age, PVs before and after castration, and volume ratios are presented in Table 2.

### Serum CRP concentrations

Serum CRP concentrations remained below the clinical threshold of 30 mg/L across all groups, including the control, pre-castration, and post-castration BPH groups. No statistically significant differences in CRP concentrations were observed among the groups.

### Serum A1BG concentrations

**Figure 2:**
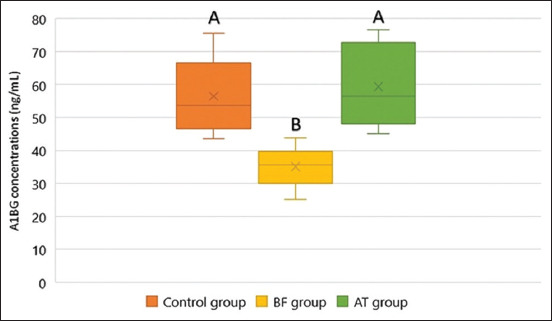
Box plot illustrating serum alpha-1B glycoprotein (A1BG) concentrations in the control, before castration (BF), and after castration (AT) groups. A1BG levels were significantly lower in the BF group than in the control and AT groups. No significant differences were observed between the control and AT groups. Differences between groups indicated by distinct superscript letters were considered statistically significant (p < 0.01).

Comparative analysis of serum A1BG concentrations among the groups is illustrated in Figure 2 and detailed in Table 3. Serum A1BG levels were significantly lower in the BPH-before-castration (BF) group (35.12 ± 5.72 ng/mL) compared with the control group (56.50 ± 11.25 ng/mL, P < 0.01) and the after-castration (AT) group (59.35 ± 12.21 ng/mL, P < 0.01). No significant difference was observed between the control and AT groups (P = 0.59).

**Table 3:** Details the comparative analysis of CRP and A1BG concentrations among the control, before castration (BF), and after castration (AT) groups.

ID	Control group		ID	BPH group			

				BF group		AT group	
		
	CRP (mg/L)	A1BG (ng/mL)		CRP (mg/L)	A1BG (ng/mL)	CRP (mg/L)	A1BG (ng/mL)
CT1	<30	64.59	BP1	<30	43.72	<30	72.19
CT2		55.02	BP2		36.49		74.56
CT3		43.65	BP3		38.85		60.74
CT4		52.10	BP4		34.65		52.26
CT5		75.62	BP5		33.48		65.88
CT6		72.65	BP6		30.12		76.64
CT7		57.74	BP7		25.20		46.93
CT8		43.92	BP8		42.23		50.90
CT9		52.23	BP9		36.46		45.08
CT10		47.47	BP10		29.97		48.32
Mean ± SD		56.50 ± 11.25			35.12 ± 5.72		59.35 ± 12.21

CT=Control dog, BP=Dog with benign prostatic hyperplasia, BPH=Benign prostatic hyperplasia, A1BG=Alpha-1B glycoprotein, CRP=C-reactive protein, SD=Standard deviation

### Correlations between serum A1BG concentrations and age

**Figure 3:**
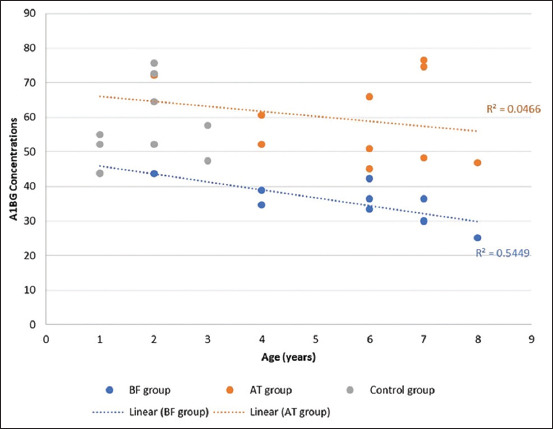
Scatter plot with a trend line shows the correlation coefficient between serum alpha-1B glycoprotein (A1BG) concentration and age among the control, before castration (BF), and after castration (AT) groups. A significant negative correlation is found only in the BF group, suggesting a potential association between age and A1BG levels in untreated benign prostatic hyperplasia dogs.

Spearman’s correlation analysis revealed a significant inverse correlation between serum A1BG concentration and age in the BF group (r = −0.74, p = 0.02) (Figure 3). In contrast, no significant correlation was observed between serum A1BG concentration and age in the AT group (r = −0.14, p = 0.71).

## DISCUSSION

### PV reduction following castration

To the best of our knowledge, this study is the first to evaluate serum CRP and A1BG concentrations in dogs with BPH before and after castration, the current gold standard treatment for this condition. Our findings confirmed a significant reduction in PV post-castration, consistent with a previous report by Schrank and Romagnoli [14], which indicated that decreased testosterone levels promote pronounced prostatic involution. Castration reduces DHT production, a critical androgen for prostatic growth, thereby inducing apoptosis in stromal and epithelial cells and ameliorating BPH-related symptoms [5]. Previous studies by Smith [26] and Spada *et al*. [27] have demonstrated that castration can decrease prostate size by up to 70%, with reductions typically beginning within 7–14 days and completing within 4 months post-surgery. Early post-castration prostatic shrinkage is closely associated with decreased testosterone levels, prostate atrophy, and alterations in prostatic artery hemodynamics [28]. In addition, castration reduces the risk of secondary complications, including prostatic infections, cyst formation, and malignant transformation [14]. Although medical treatments, such as finasteride, also reduce PV, they act more slowly and may be associated with adverse effects, including sexual dysfunction during long-term use [28, 29]. These observations underscore the importance of further elucidating BPH pathophysiology and support the utility of novel biomarkers for diagnosis and therapeutic monitoring.

### Serum A1BG as a potential biomarker

This study provides the first comprehensive evaluation of serum A1BG concentrations in dogs with BPH before and after castration, thereby advancing the field of canine prostatic biomarker research. A significantly lower serum A1BG concentration was observed in the BPH-before castration (BF) group compared to healthy controls, highlighting a potential involvement of A1BG in BPH pathogenesis. These results align with a prior proteomic study by Ploypetch *et al*. [22] that identified reduced expression of a candidate peptide predicted to be A1BG in dogs with BPH. Notably, no significant differences in serum A1BG levels were observed between the control and after-castration (AT) groups, suggesting biochemical normalization following castration. This finding is consistent with the well-documented effects of castration, including reductions in PV and the resolution of pathological processes [14].

A1BG is increasingly recognized as a biomarker of diagnostic and therapeutic relevance in inflammatory and immune-modulatory conditions [30]. In the context of BPH, decreased A1BG levels observed in the BF group may reflect an active role in modulating local immune responses during chronic prostatic inflammation. Chronic inflammation is a hallmark of BPH, and A1BG may participate in the regulation of localized immune processes, as previously suggested in veterinary [22] and human [30] studies. The normalization of A1BG concentrations post-castration underscores its potential utility as a marker of therapeutic efficacy, reflecting the resolution of inflammatory and hormonal disturbances.

### Correlation between age and serum A1BG concentrations

A significant inverse correlation was identified between serum A1BG concentrations and age in the BF group, suggesting that age-related processes, such as chronic low-grade inflammation (“inflammaging”), may influence A1BG expression. Aging in dogs is characterized by a progressive elevation in systemic pro-inflammatory markers, a phenomenon implicated in the pathogenesis of numerous age-associated diseases, including cardiovascular disorders, arthritis, and neoplasia [31, 32]. Furthermore, aging is associated with hormonal imbalances, particularly elevated DHT levels, which contribute to prostatic growth and inflammation [33]. These hormonal and inflammatory alterations may indirectly affect A1BG regulation through inflammatory and androgen receptor signaling pathways. The absence of an age-A1BG correlation in the post-castration group supports the hypothesis that castration mitigates these effects by restoring hormonal balance and reducing chronic inflammation [34].

### Evaluation of serum CRP concentrations

Serum CRP concentrations did not differ signifi-cantly between the groups, suggesting that CRP is not a reliable marker of localized, low-grade prostatic inflammation associated with BPH. This finding is consistent with a study by Lehrer *et al*. [19] in humans, where chronic prostatic inflammation often remains undetected by systemic CRP assays. In contrast, CRP levels typically rise markedly during acute or systemic inflammatory processes [16]. Although elevated CRP concentrations have demonstrated prognostic relevance in prostate cancer, serving as an indicator of cancer-specific survival [35], its limited sensitivity for detecting chronic, localized inflammation in BPH reduces its diagnostic utility in this context. These findings underscore the need for alternative biomarkers, such as A1BG, in the diagnosis and monitoring of canine BPH.

## CONCLUSION

This study demonstrated that serum A1BG serves as a promising biomarker for the diagnosis and therapeutic monitoring of BPH in dogs. Serum A1BG concentrations were significantly lower in dogs with BPH before castration compared to healthy controls, and normalization of A1BG levels was observed following surgical intervention. In contrast, CRP concentrations remained within the normal range and did not differ significantly across groups, indicating limited diagnostic utility for detecting localized prostatic inflammation. A significant inverse correlation between serum A1BG concentrations and age in BPH-affected dogs before castration further suggests an interaction between age-related inflammatory processes and A1BG expression.

The practical implications of these findings are considerable. Incorporating serum A1BG measurement into clinical diagnostic workflows could enhance the early detection, differentiation, and monitoring of BPH in canine patients, offering a noninvasive and accessible alternative to imaging and invasive tissue sampling. Monitoring A1BG levels post-castration could also aid in evaluating treatment response and disease resolution, ultimately improving clinical decision-making and patient outcomes.

The primary strength of this study lies in its comprehensive evaluation of A1BG dynamics before and after castration, which has not previously been investigated in canine BPH. By coupling biomarker analysis with clinical assessments and PV measurements, the study provides robust preliminary evidence supporting the integration of A1BG as a clinically informative biomarker. Furthermore, the study utilized rigorous methodological standards, including validated assays, ultrasonographic confirmation of BPH, and appropriate statistical analyses, enhancing the reliability and reproducibility of the findings.

Despite the robustness of the findings, this study has certain limitations. Although the study was statistically powered, the relatively small sample size may limit the generalizability of the results. Furthermore, the 1-month post-castration follow-up period may not fully capture long-term biomarker dynamics or the persistence of biochemical changes. Although efforts were made to minimize confounding factors, age-related disparities between groups could have influenced the results.

Future research should address these limitations by enrolling larger, more diverse canine populations representing various breeds and age groups. Longitudinal studies with extended follow-up periods are warranted to elucidate the temporal dynamics of A1BG concentrations and their clinical implications. Specifically, monitoring A1BG levels over a period of 6 months to 1-year post-castration or in dogs undergoing medical management for BPH would provide valuable insights. In addition, the implementation of high-sensitivity biomarker assays, similar to those utilized in human medicine, may enhance the detection of subtle variations in biomarker expression [36].

## DATA AVAILABILITY

All the generated data are included in the manuscript.

## AUTHORS’ CONTRIBUTIONS

NP, GW, and SP: Conceptualization, methodology, formal analysis, investigation, data curation, funding acquisition, writing-original draft preparation, and writing-review and editing. PP, KU, KN, RS, and TT: Methodology, formal analysis, and writing-original draft preparation. All authors have read and agreed to the publication of the final version of the manuscript.
